# An Inverse Optimization Method for the Parameter Determination of the High-Temperature Damage Model and High-Temperature Damage Graph of Ti6Al4V Alloy

**DOI:** 10.3390/ma16134770

**Published:** 2023-07-01

**Authors:** Xuewen Chen, Zhen Yang, Bo Zhang, Jiawei Sun, Zhiyi Su, Yiran Mao

**Affiliations:** School of Materials Science and Engineering, Henan University of Science and Technology, 263 Kaiyuan Avenue, Luoyang 471023, China; 13544881895@163.com (Z.Y.); zhangbo32103@126.com (B.Z.); 210321020208@stu.haust.edu.cn (J.S.); 210321020235@stu.haust.edu.cn (Z.S.); 220320020200@stu.haust.edu.cn (Y.M.)

**Keywords:** Ti6Al4V alloy, high-temperature damage model, optimization reverse, damage graph

## Abstract

Ti6AL4V alloy is widely used in the biomedical and energy vehicle industries, among others. Ti6Al4V alloy cannot be fabricated at ambient temperatures; hence, it requires hot forming. However, this method is susceptible to crack defects. The crack defect problem of Ti6AL4V alloy in the hot-forming process cannot be ignored, so we must develop a precise hot-forming damage prediction model. In this study, three high-temperature damage models of Ti6Al4V alloy were developed, considering the temperature and strain rate. These models were derived from the normalized Cockcroft and Latham (NCL), Oyane, and Rice and Tracey (RT) damage models. The damage parameters of the models were identified using a genetic algorithm combined with finite element simulation. The force accumulation error of the Ti6AL4V alloy specimen, which was obtained from a simulated thermal tensile test and an actual test, was used as an optimization target function. Then, the damage parameters were optimized using the genetic algorithm until the target function reached the minimum value. Finally, the optimal damage model parameter was obtained. Through program development, the three high-temperature damage models established in this paper were embedded into Forge^®^ NxT 2.1 finite element software. The simulated thermal tensile test of Ti6AL4V alloy was performed at a temperature of 800–1000 °C and a strain rate of 0.01–5 s^−1^. The simulated and actual fracture displacements of the tensile specimens were compared. The correlation coefficients (R) were calculated, which were 0.997, 0.951, and 0.912. Of the high-temperature damage models, the normalized Cockcroft and Latham high-temperature damage model had higher accuracy in predicting crack defects of Ti6Al4V alloy during the hot-forming process. Finally, a fracture strain graph and a high-temperature damage graph of Ti6Al4V alloy were constructed. The Ti6Al4V alloy damage evolution and thermal formability were analyzed in relation to the temperature and strain rate.

## 1. Introduction

Ti6Al4V is a titanium alloy with a two-phase structure of α–β, which has the advantage of high strength, hardness, excellent fatigue resistance, biocompatibility, and low density [1,2]. At room temperature, Ti6Al4V alloy has a hexagonal close-packed (HCP) α phase, which only has three slip systems. This results in poor plasticity, high hardness, and high deformation resistance [3]. The α–β phase transition temperature of Ti6Al4V alloy is between 980 °C and 990 °C. The body-centered cubic structure (BCC) of the β phase has 12 slip systems [4]. Therefore, the strength and hardness of Ti6Al4V alloy will significantly decrease at high temperatures, while the plasticity and workability will improve. However, Ti6Al4V alloy cracks easily during hot processing because the α phase still exists in the hot-forming process. When the crack expansion is severe, the blank will be completely scrapped. Therefore, for the purpose of predicting the initiation of cracks accurately and effectively controlling crack defects in hot deformation, it is important to develop an accurate high-temperature damage model for Ti6Al4V alloy.

With the development of damage mechanics, numerous researchers have conducted relevant studies on the failure modes and damage mechanisms of materials. Based on different damage mechanisms, classical damage models were constructed [5]. Classical damage models have been widely used because of their concise form and fewer parameters [6]. Classical damage models use the critical damage value as the criterion; when the cumulative damage value reaches the critical value, the material will crack. Freudenthal [7] first proposed the Freudenthal model in 1950, which used the material strain energy to determine whether damage would occur. As soon as the strain energy reaches the critical level, the material will be destroyed. Cockcroft and Latham [8] proposed the Cockcroft and Latham (CL) model, which assumed that the maximum tensile stress is the main reason for metal failure. Rice and Tracey [9] discussed the ductile failure of materials containing independent spherical defects in the triaxial stress and proposed the Rice and Tracey (RT) model. This model can predict the initiation of cracks and the direction of crack propagation. Considering the first principal stress and the equivalent stress, Oh and Kobayashi et al. [10] combined the void growth theory with the CL model and put forward the normalized Cockcroft and Latham (NCL) model. Brozzo [11] considered the influence of hydrostatic stress on metal damage. The Brozzo model was developed based on the CL model. Oyane [12] assumed that the second-phase particles caused the crack defect in the metal deformation process and proposed the Oyane model, which considers stress triaxiality.

Classical damage models have the advantages of concise mathematical expressions, high computing efficiency, and strong reliability. The damage parameters can be determined through a simple tensile test. Therefore, these models have been widely applied and verified in enterprise production, usually combined with finite element software to predict cold-forming crack damage in actual industrial production. HariKrishna et al. [13] investigated the toughness fracture of AA2014 cast alloy, which is embedded with fly ash composite material, during room-temperature upsetting by using the Oyane damage model. Hwang et al. [14] studied the fracture mechanism of hydraulic punching by using the NCL damage model and optimized the punch shape in the hydraulic punching process of sPFC590Y carbon steel tubes. Li et al. [15] used the Abaqus with eight commonly used uncoupled damage models to predict the fracture of thin-walled metal plates during room-temperature spinning forming. The results showed that the Oyane and Oh damage models had a more accurate capability to predict fracture in spinning processes. Mastrone et al. [16] calibrated the Rice and Tracey damage model for aluminum alloy cold forming and validated it using finite element analysis in punch testing. There was consistency between the test observations and simulation results.

However, traditional damage models do not take the temperature and strain rate into account. When metal is formed at high temperatures, various temperatures and strain rates will lead to comprehensive thermodynamic phenomena, such as grain growth, dynamic recrystallization, and phase transformation [17]. The comprehensive thermo-mechanical effects in the hot-working process are ultimately reflected in the macroscopic properties of the material. For instance, the occurrence of the material softening effect makes the material more ductile and has a significant impact on the forming of cracks in the material. Therefore, traditional damage models cannot be directly applied to predict damage during the hot deformation of metals. Due to the fact that traditional damage models are unable to predict the high-temperature damage evolution of materials, it is necessary to develop corresponding high-temperature damage models that consider the temperature and strain rate. Jia [18] believed that the fracture strain and critical damage value of materials are linearly correlated with the Zener–Hollomon parameter. He used the modified Freudenthal damage model to investigate the thermal damage behavior of AZ31B. Zhang et al. [19] coupled the temperature, strain rate, and critical damage to modify the traditional NCL damage model and applied it with finite element software to obtain the damage parameters in the forming process of nickel-based alloys. Cao et al. [20] obtained the Brozzo critical damage value by conducting isothermal compression tests and finite element simulation tests at low strain rates and high temperatures. The Brozzo-modified damage model of Ti-47Al-2Nb-2Cr alloy was established by coupling the critical damage value with the Zener–Hollomon parameter.

The accuracy of the identification of damage model parameters is crucial for the precise prediction of the material’s damage behavior during hot deformation. The methods for determining damage model parameters mainly include experimental methods, numerical derivation methods, analytical methods, and indirect measurement methods [21]. However, most traditional methods are based on a large amount of experimental analysis and complex characterization methods, and as the temperature rises or the strain rate changes, some damage parameters become more difficult to identify. On the other hand, it is difficult to guarantee the accuracy of identification. With the development of computer technology and mathematical algorithms, computer-based parameter inversion has emerged as a new method for parameter determination. Parameter inversion techniques are based on material properties such as stress–strain curves or force–displacement curves. After selecting appropriate algorithms and objective functions, the finite element simulation is iterated until the parameters that best match the numerical calculation and experimental properties are found [22]. Muñoz-Rojas et al. [23] used genetic algorithms to identify the parameters of the GTN model and elastic–plastic parameters in the work-hardening model for 7055 aluminum alloy under 400 °C and 1 mm/min tensile rate. Sprave and Menzel [24] used the force–displacement data obtained from simulations and experiments to construct the objective function for DP800 dual-phase steel notch specimens. They used the linear programming–simplex method to identify the parameters of the gradient-enhanced ductility damage model for DP800 steel and determine the optimal parameter set for the model. Tang [25] compared the load–displacement curves of 22MnB5 obtained from experiments and simulations and reversed the parameters of the Lemaitre damage model during the thermal stamping process.

The damage graph of materials, covering the related fields of continuum damage mechanics, fracture criteria, and material fatigue failure, can intuitively reflect the influence of various factors on materials’ forming performance. It provides a reference for evaluating the mechanical properties of materials and preliminarily selecting the material processing parameters. Therefore, many scholars have used the damage graph as a tool to investigate the damage behavior of materials and optimize craft parameters. Fu et al. [26] constructed a comparison chart of fracture strain–size–fracture energy based on non-coupled damage models such as Freudenthal, NCL, Brozzo, and Oyane to investigate the relationship between the damage behavior and size effect in multiphase alloy brass C3602. They found that the Freudenthal criterion was more suitable for analyzing the damage process of C3602 in micro-forming. González et al. [27] conducted a comparative study of toughness failure models for the unidirectional drawing process of Al-2011 aluminum alloy. They constructed the critical damage value reduction ratio graphs based on the RT, CL, and Brozzo damage models to obtain the damage values and determined the optimal range of reduction parameters from the graph. Lou et al. [28] selected the Brozzo, Clift, and CL damage models based on Al2024-T351 and constructed fracture strain–stress triaxiality and Lode angle fracture surface charts under the corresponding model predictions. The results showed that all toughness damage models, except for the Clift damage model, displayed a negative correlation between fracture strain and stress triaxiality, and all toughness damage models predicted lower material forming properties under simple shear stress than under simple tensile stress.

An accurate damage model is crucial for reducing the cost of trial and error during production, and for improving the hot-forming production process of Ti6Al4V alloy. At present, there are many damage models that can be used in sheet forming. According to the stress state and damage mechanism of the sheet-forming process, high-temperature damage models for Ti6Al4V alloy were established using NCL, RT, and Oyane damage models, and the high-temperature damage model with the highest prediction accuracy was selected. Ti6Al4V alloy specimens were subjected to thermal tensile tests on Gleeble-1500D at temperatures from 800 to 1000 °C and strain rates from 0.01 to 5 s^−1^. Based on the finite element simulation technology and optimization algorithms, a method for optimizing and inversely determining damage parameters was proposed. During secondary development, the high-temperature damage models were compiled into Forge^®^, and numerical simulations were performed for thermal tensile tests of Ti6Al4V alloy. The fracture displacements of the simulated and actual alloys were compared. Subsequently, the correlation coefficient (R) was calculated. The optimal prediction model for Ti6Al4V alloy hot-forming damage was determined. Ti6Al4V alloy’s high-temperature damage graph and fracture strain graph were established for studying the temperature and strain rate effect on Ti6Al4V alloy’s damage evolution further. The damage behavior and hot-forming properties of Ti6Al4V alloy under various temperatures and strain rates were analyzed.

## 2. Materials and Methods

**Figure 1:**
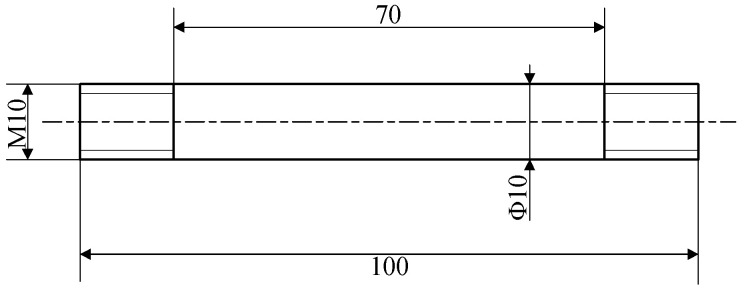
The size of the thermal tensile sample.

**Figure 2:**
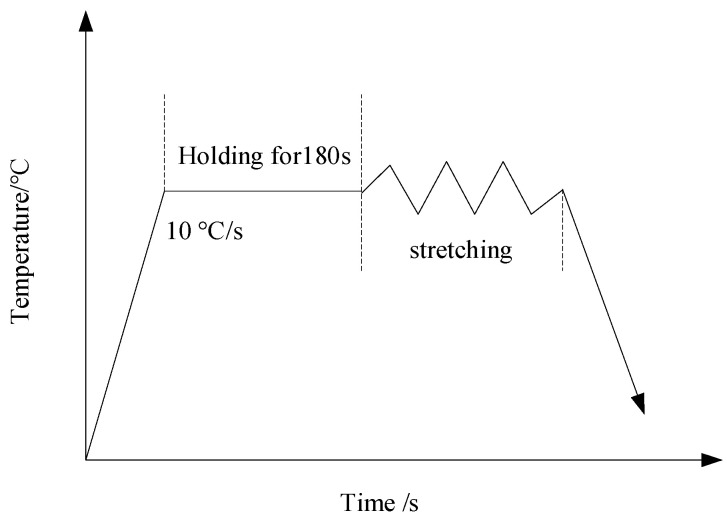
Technological scheme of thermal tensile test.

The testing material was forging Ti6Al4V alloy. Table 1 shows its chemical compositions. Figure 1 shows the shape and dimensions of the thermal tensile cylindrical specimen, which was prepared in accordance with ASTM E8/E8M-2016a. In order to describe Ti6Al4V alloy’s hot-forming flow stress and damage behavior, thermal tensile tests should be conducted. Based on the Ti6Al4V alloy’s commonly used temperature and strain rate in actual production, 5 sets of temperatures (800 °C, 850 °C, 900 °C, 950 °C, and 1000 °C) and 4 sets of strain rates (0.01 s^−1^, 0.1 s^−1^, 1 s^−1^, and 5 s^−1^) were selected, resulting in 20 sets of high-temperature tensile tests. The process scheme for the high-temperature tensile tests is shown in Figure 2. The thermal tensile test was conducted on the Gleeble-1500D. At the gauge distance position, the specimens were heated to the target temperature at 10 °C per second before the tensile test, and the temperature was held for 180 s to eliminate the temperature gradient.

## 3. Thermal Tensile Test Results of Ti6Al4V Alloy

**Figure 3:**
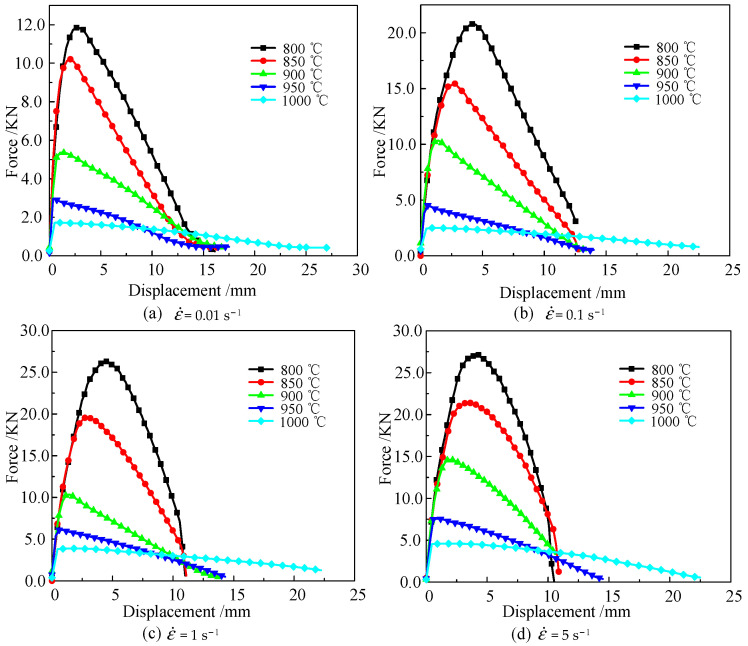
The force–displacement curve of Ti6Al4V alloy.

The force–displacement curves of Ti6Al4V alloy are shown in Figure 3. The elastic deformation stage was not significant during the Ti6Al4V alloy’s thermal tensile process. The yield strength of the alloy declined under high temperatures, resulting in a short elastic deformation stage. As the amount of elongation increased, plastic deformation became the main mechanism. At temperatures of 800 °C, 850 °C, 900 °C, 950 °C, and 1000 °C and strain rates of 0.01 s^−1^, the Ti6Al4V alloy’s peak tensile force was 11.87, 10.21, 5.36, 2.97, and 1.71 KN, respectively. When the strain rate was constant, the maximum tensile force had a negative correlation with the temperature. When the temperature was 800 °C, and the strain rate was 0.01 s^−1^, 0.1 s^−1^, 1 s^−1^, and 5 s^−1^, the maximum tensile force was 11.87, 20.80, 26.85, and 27.11 KN, respectively. Compared with the peak force at 800 °C/0.01 s^−1^, the peak tensile force at the strain rate of 5 s^−1^ increased by 128.39%. Thus, when the temperature was constant, the peak tensile force had a positive correlation with the strain rate.

The fundamental reason why the temperature and strain rate influenced the Ti6Al4V alloy’s deformation resistance and fracture displacement is the comprehensive effect of dynamic recovery and recrystallization during the hot-forming process. At lower temperatures, Ti6Al4V alloy’s dynamic recrystallization was suppressed. As deformation continued, the dislocation density continued to increase, leading to an increase in the resistance of dislocation motion and deformation [29]. When the Ti6Al4V alloy’s temperature was heated to the dynamic recrystallization temperatures, the dynamic recrystallization effect intensified with an increase in the strain rate. However, the increased rate of dislocation density also continued this trend, which led to the work-hardening effect of the Ti6Al4V alloy being greater than the softening effect. However, this trend diminished with the continuous increase in temperature. At higher temperatures, the enhanced dynamic recrystallization effect offsets the increase in the work-hardening effect, leading to a decrease in the dominance of the work-hardening effect. When the temperature was 800 °C and the strain rate increased from 0.01 s^−1^ to 0.1 s^−1^, the peak force increased by 10.60 KN. When the strain rate improved from 1 s^−1^ to 5 s^−1^, the peak force only increased by 1.78 KN. Thus, the Ti6Al4V alloy’s softening effect in the thermal deformation process was weaker than the work-hardening effect. However, this trend gradually diminished with the increase in temperature.

The deformation temperature also significantly affected the fracture displacement of the Ti6Al4V alloy. At 800 °C/5 s^−1^, the fracture displacement was 10.623 mm. When the temperature increased to 1000 °C, the fracture displacement increased to 22.797 mm. The strain rate also affected the fracture displacement. When the deformation temperature was 1000 °C, and the strain rate was reduced to 0.01 s^−1^, the fracture displacement of the material also decreased to 16.019 mm. Thus, the fracture displacement of Ti6Al4V alloy is positively correlated with temperature and negatively correlated with strain rate.

## 4. Modification and Parameter Inverse Optimization of Ti6Al4V alloy Damage Models

### 4.1. Thermal Deformation Constitutive of Ti6Al4V Alloy

Due to the fact that the temperature and strain rate significantly affect the Ti6Al4V’s flow stress behavior, a constitutive model that is independent of the strain rate and temperature cannot characterize the flow stress behavior of Ti6Al4V during hot-forming processes. Therefore, in this paper, the Hansel–Spittel constitutive model was used to accurately characterize the Ti6Al4V alloy’s flow stress behavior at high temperatures, as shown in Equation (1). The parameters were obtained from previous studies [30] and are listed in Table 2.

(1) $$\sigma =A\cdot e^{m_{1}T}\cdot \varepsilon^{m_{2}}\cdot {\dot{\varepsilon }}^{m_{3}}\cdot e^{\frac{m_{4}}{\varepsilon }}\cdot {\left(1+\varepsilon \right)}^{m_{5}T}\cdot e^{m_{7}\varepsilon }\cdot {\dot{\varepsilon }}^{m_{8}T}\cdot T^{m_{9}}$$

where $m_{1}$ is the temperature-related coefficient, $m_{2}$ is the strain-hardening exponent, $m_{3}$ is the strain-rate-hardening exponent, $m_{4}$ is the strain-softening coefficient, $m_{5}$ is the temperature–strain coupling coefficient, $m_{7}$ is the strain-strengthening coefficient, $m_{8}$ is the strain rate–temperature coupling coefficient, $m_{9}$ is temperature-strengthening exponent, *A* is a material constant, σ is the flow stress (MPa), T is the temperature (K), $\dot{\varepsilon }$ is the strain rate (s^−1^), and ε is the plastic strain.

### 4.2. High-Temperature Damage Model of Ti6Al4V Alloy

The classical damage model can be represented as the stress function integrating with strain, as shown in Equation (2).

(2) $$C=\int_{0}^{{\bar{\varepsilon }}_{f}}f\left(\sigma_{ij}\right)d\bar{\varepsilon }$$

where *C* is the cumulative damage value, ${\bar{\varepsilon }}_{f}$ is the fracture strain, and $f\left(\sigma_{ij}\right)$ is the stress-related function.

An NCL model is defined as the strain energy function of the maximum principal stress, normalized with effective stress. Thus, it is suitable for damage prediction dominated by tensile stress. The Oyane model is derived from the plasticity theory of porous materials, which is suitable for fracture forms caused by pore evolution [31]. The RT model is defined as a function of the normalized mean stress [32], which is suitable for predicting the blanking behavior. In order to explore the damage mode of Ti6AL4V alloys during the plate-rolling process, the above three damage models were selected. The NCL, Oyane, and RT damage models are shown in Table 3.

Traditional damage models are commonly used for predicting damage during metal cold forming. However, the damage and fracture behavior of Ti6Al4V alloys varies with changes in temperature and strain rate. Thus, it is necessary to modify the traditional model. Based on the model’s definition and form, the traditional damage model can be modified using Equation (3).

(3) $$K=\frac{C}{D}=\int_{0}^{{\bar{\varepsilon }}_{f}}\frac{f\left(\sigma_{ij}\right)}{S(T,\dot{\bar{\varepsilon }})}d\bar{\varepsilon }$$

where the material will undergo ductile fracture when *K* = 1; *D* is the critical damage value; and $S(T,\dot{\bar{\varepsilon }})$ is a function to describe *D* changes with the temperature and the strain rate.

Once the stress function $f\left(\sigma_{ij}\right)$ is determined, the form of the function $S(T,\dot{\bar{\varepsilon }})$ must be determined to calculate the critical damage value. The Zener–Hollomon parameter can reflect the effect of the strain rate and temperature during the alloy hot-forming process [33].

(4) $$Z=\dot{\varepsilon }\exp\left[Q/RgT\right]$$

where Q is the thermal activation energy, $\dot{\varepsilon }$ is the plastic strain rate, Rg is the gas constant, and T is the Kelvin temperature.

Therefore, the function S can be expressed in $S(T,\dot{\bar{\varepsilon }})=S(Z)$. The damage model can be expressed as Equation (5). Finally, a modified form of the damage model can be obtained, as shown in Table 4.

(5) $$K=\int_{0}^{{\bar{\varepsilon }}_{f}}\frac{f\left(\sigma_{ij}\right)}{S(T,\dot{\bar{\varepsilon }})}d\bar{\varepsilon }=\int_{0}^{{\bar{\varepsilon }}_{f}}\frac{f\left(\sigma_{ij}\right)}{S(Z)}d\bar{\varepsilon }$$

### 4.3. Parameter Identification of High-Temperature Damage Model for Ti6Al4V Alloy

**Figure 4:**
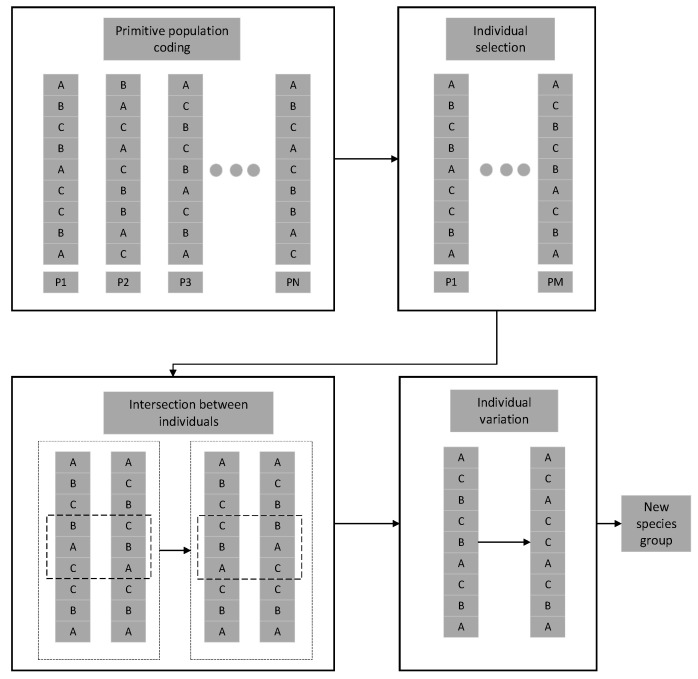
Principles of genetic algorithms.

The GA (genetic algorithm) method was first proposed by J.H. Holland in 1992. It is a stochastic global search optimization method named after Darwin’s theory of evolution. The core concept of the algorithm is derived from the concepts of genetics, mutation, natural selection, and hybridization in the theory of evolution [34]. This algorithm uses mathematical methods and computer simulation calculations to convert problem-solving processes into processes similar to chromosome gene crossbreeding and mutation in biological evolution. Genetic algorithms are commonly used for single-objective optimization problems. The principles of numerical encoding, crossover, and mutation are shown in Figure 4. The initial target values need to be encoded with a specific string length, population size, crossover rate, and mutation rate within their value ranges. After encoding, the objective function is used to compute the individual’s fitness value. Later, the selection operator is used to screen individuality, and the selected individuals are subjected to crossover and mutation to obtain new individuals. After all the individuals have gone through the above process, they are combined into a new population for the next iteration.

In order to accurately obtain the parameters of high-temperature damage models in different conditions, a combination of the genetic algorithm method and the finite element method was used to identify the parameters. In the inverse optimization design, the critical damage value was first determined as the damage parameter, and the cumulative error of fracture displacement between the simulated and the actual specimens was selected as the target function. Then, the finite element model was established in the Forge^®^ finite element software for tensile tests. In the simulation process of the thermal tensile test, the damage parameters were updated and adjusted according to the genetic algorithm. Finally, when the target function reached the minimum convergence range, the corresponding damage parameter was optimal.

**Figure 5:**
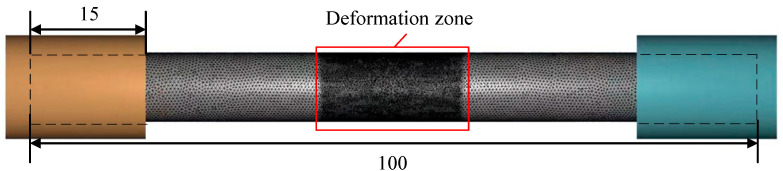
Tensile geometric model of TC4 titanium alloy.

Localized meshing technology was used to improve the computational accuracy and speed up the calculation time. Briefly, a 0.1 mm mesh size was used in the center tensile deformation areas, and a 0.5 mm mesh size was used in other areas, as shown in Figure 5. To simulate the situation in which the threads of the tensile specimen get stuck in the clamping fixture, the tensile specimen was extended 15 mm into the clamping fixture. Based on the actual experimental conditions, the deformation area of the sample was heated to a specific temperature in an adiabatic condition. The thermal conductivity of the sample was 7.955 W/m·°C [35], and the conversion efficiency from plastic work to heat was 0.88 [36]. The two ends of the sample were clamped into the fixture and stretched at an actual stretching speed.

**Figure 6:**
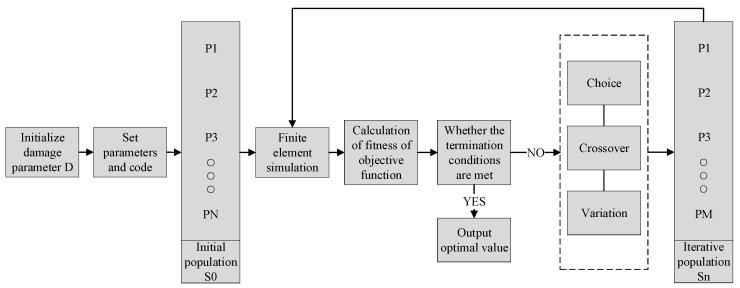
Flowchart of identification by the inverse technique.

The process of optimizing reverse design is shown in Figure 6. The target function for this process was the cumulative error between the simulated and actual force values, as shown in Equation (6). As the target function value approached zero, the simulation results became increasingly closer to the actual test results.

In single-objective optimization solutions, the population size of the genetic algorithm has an impact on the iteration result and iteration time. The population size of the genetic algorithm should empirically be between 16 and 150. The crossover rate is generally in the range of 0.6–0.9, and a larger crossover rate improves the emergence probability and the overall convergence speed of new individuals in each generation. A larger crossover rate facilitates the elimination of promising individuals that were prematurely eliminated in the iterative process. The accuracy of the final result with a larger crossover rate was not as good as that with a smaller crossover rate. The mutation rate is generally in the range of 0.001–0.1, and a lower mutation rate leads to a small number of new individuals, but the overall convergence is more stable [23]. The genetic algorithm parameters were chosen in order to improve the reverse recovery speed of the damage parameters and ensure solution accuracy; these parameters are shown in Table 5.

(6) $$\varphi =\frac{\sum_{i}\left[{\left(y_{i}{}^{\exp}-y_{i}{}^{num}\right)}^{2}\right]}{\sum_{i}\left[{\left(y_{i}{}^{\exp}\right)}^{2}\right]}$$

where $y_{i}{}^{\exp}$ is the force of the *i*-th data point in tests, and $y_{i}{}^{num}$ is the force of the *i*-th data point in simulations.

Ti6Al4V alloy’s optimal reverse results of the critical damage value of the NCL, Oyane, and RT damage models in different conditions are shown in Table 6.

**Figure 7:**
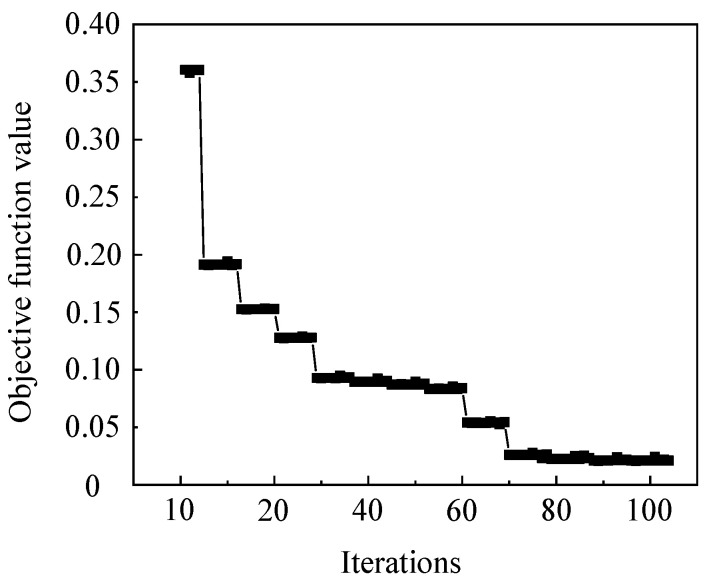
Convergence curve of optimization algorithm for strain rate 5 s^−1^ at 800 °C.

**Figure 8:**
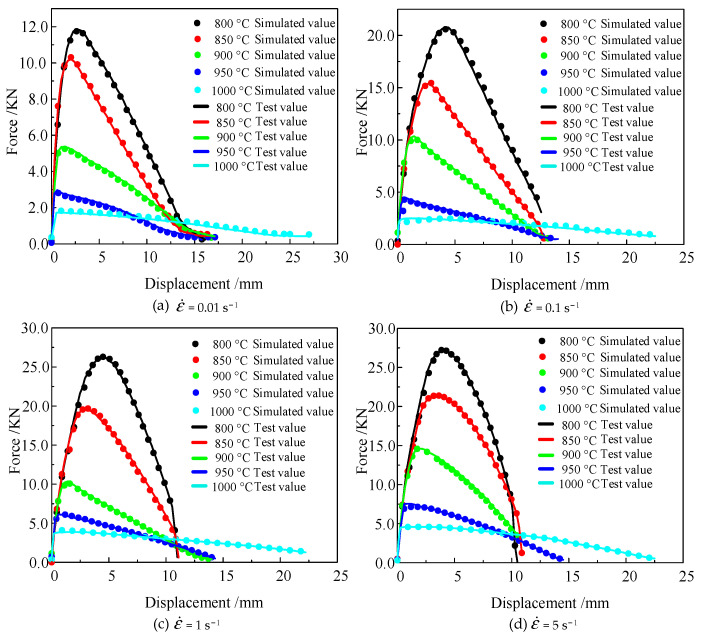
Comparison between the experimental data and the simulation results based on the NCL damage model.

Figure 7 shows the optimization convergence curve of critical damage values for Ti6Al4V alloy based on the NCL damage model at 800 °C/5 s^−1^. The objective function converges to the target range after 106 iterations. Figure 8 shows the comparison of the simulation and reality force–displacement curves, which use the reversed critical damage value based on the NCL damage model. It can be seen that the curves of the simulated and real scenarios are in good agreement. The results indicate that the damage-parameter-optimized inversion method as a combination of genetic algorithm and finite element methods has a high convergence speed, and the required accurate damage parameters can be obtained after a finite number of iterations. Equation (6) was used to calculate the different damage models’ cumulative error of the simulated and actual force–displacement curves at 800–1000 °C and 0.01–5 s^−1^, taking the average value. The average cumulative error is shown in Table 7.

One of the parameters determined in the Zener–Hollomon equation is the activation energy *Q*. By taking the logarithm of Equations (4) and (7)–(9) and performing linear fitting [37], the parameter *Q* can be obtained.

(7) $$\dot{\varepsilon }=A_{1}[\sinh{\left(\alpha \sigma \right)}^{n_{2}}\exp(-Q/RgT)]$$

(8) $$\dot{\varepsilon }=A_{2}\sigma^{n_{1}}\exp(-Q/RgT)$$

(9) $$\dot{\varepsilon }=A_{3}\exp(\beta \sigma )\exp(-Q/RgT)$$

Finally, the activation energy *Q* was obtained as 809,133.87 J·mol^−1^. Thus, the Zener–Hollomon equation can be expressed as follows:

(10) $$Z=\dot{\varepsilon }\exp(\frac{809133.87}{RgT})$$

As shown in Table 8, the Zener–Hollomon parameter was calculated according to the high-temperature tensile test data of Ti6Al4V alloy.

**Figure 9:**
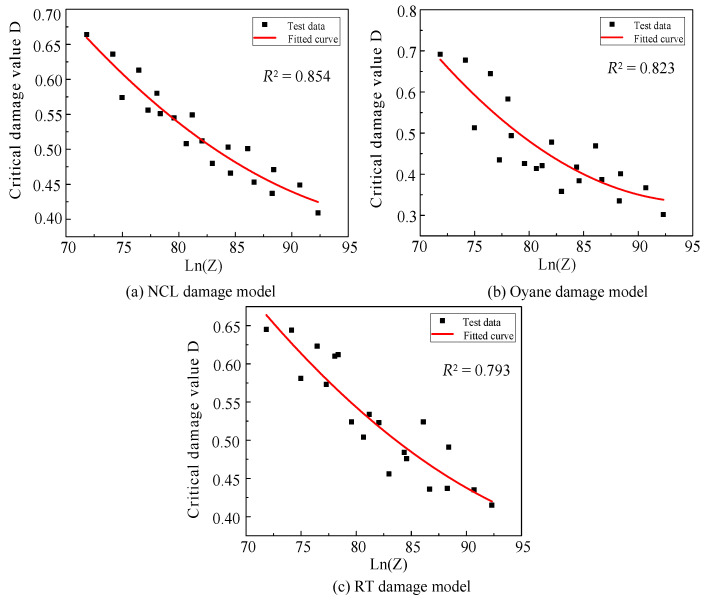
The fitting curve of the critical damage value of different damage models.

In order to couple the damage parameter with the *Z* parameter, the second-order polynomial was used to fit $\ln^{Z}$ with the damage parameters in Table 6, and the fitting curves are shown in Figure 9.

(11) $$S_{1}(Z)=3.3799-0.05837\ln^{Z}+0.0002855(\ln^{Z}{)}^{2}$$

(12) $$S_{2}(Z)=6.00572-0.1189\ln^{Z}+0.0006228{\left(\ln^{Z}\right)}^{2}$$

(13) $$S_{3}(Z)=3.05469-0.04989\ln^{Z}+0.0002312(\ln^{Z}{)}^{2}$$

Functions (11)–(13) were, respectively, substituted into Equation (5). Finally, the Ti6Al4V alloy’s NCL high-temperature damage model is as follows:

(14) $$\left\{ \begin{array}{l} K=\int_{0}^{\varepsilon }\frac{\sigma_{1}/\bar{\sigma }}{3.3799-0.05837\ln Z+0.0002855(\ln Z{)}^{2}}d\bar{\varepsilon }\\ Z=\dot{\varepsilon }\exp(\frac{809133.87}{RT}) \end{array}\right.$$

The Oyane high-temperature damage model is as follows:

(15) $$\left\{ \begin{array}{l} K=\int_{0}^{\varepsilon }\frac{1+\alpha \sigma_{1}/\bar{\sigma }}{6.00572-0.1189\ln Z+0.0006228{\left(\ln Z\right)}^{2}}d\bar{\varepsilon }\\ Z=\dot{\varepsilon }\exp(\frac{809133.87}{RT}) \end{array}\right.$$

The RT high-temperature damage model is as follows:

(16) $$\left\{ \begin{array}{l} K=\int_{0}^{\varepsilon }\frac{\exp(\alpha \sigma_{m}/\bar{\sigma })}{3.05469-0.04989\ln Z+0.0002312(\ln Z{)}^{2}}d\bar{\varepsilon }\\ Z=\dot{\varepsilon }\exp(\frac{809133.87}{RT}) \end{array}\right.$$

## 5. Selection and Verification of High-Temperature Damage Model for Ti6Al4V Alloy

In order to determine the high-temperature damage model with the highest accuracy in predicting the damage behavior of Ti6Al4V alloy, all high-temperature damage models were integrated into the Forge^®^ simulation software during the secondary development. Through thermal tensile simulation tests, the simulated fracture displacement of Ti6Al4V alloy under the conditions of temperature ranging from 800 °C to 1000 °C and strain rate ranging from 0.01 s^−1^ to 5 s^−1^ was obtained.

**Figure 10:**
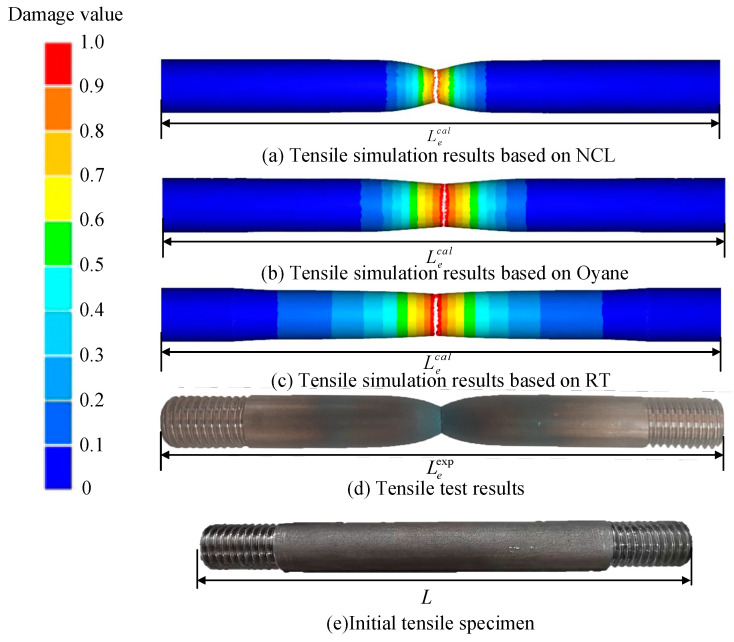
Tensile simulation results of Ti6Al4V alloy at 850 °C and 5 s^−1^.

The simulated results of Ti6Al4V alloy under the tensile conditions of 850 °C and 5 s^−1^ are shown in Figure 10. Equations (17) and (18) are used to calculate the experimental fracture displacement $\Delta L_{}^{\exp}$ and the simulated fracture displacement $\Delta L^{cal}$, respectively. The correlation coefficient (R) is calculated using Equation (19) to evaluate the correlation between the actual and the simulated results. The closer to one is the R-value, the higher the correlation between the actual results and the simulation results, and the higher the accuracy of the damage model prediction.

(17) $$\Delta L_{}^{\exp}=L_{e}^{\exp}-L$$

(18) $$\Delta L^{cal}=L_{e}^{cal}-L$$

(19) $$R=\frac{\sum_{i=1}^{N}\left(\Delta L_{i}^{\exp}-\bar{\Delta L^{\exp}}\right)\left(\Delta L_{i}^{\exp}-\bar{\Delta L^{cal}}\right)}{\sqrt{{\sum_{i=1}^{N}\left(\Delta L_{i}^{\exp}-\bar{\Delta L^{\exp}}\right)}^{2}}\sqrt{{\sum_{i=1}^{N}\left(\Delta L_{i}^{\exp}-\bar{\Delta L^{cal}}\right)}^{2}}}$$

where *L* is the initial length of the specimen, and $L_{e}^{\exp}$ and $L_{e}^{cal}$ are the lengths of the fracture specimen in the actual test and simulation. *N* is the total number of thermal tensile tests.

**Figure 11:**
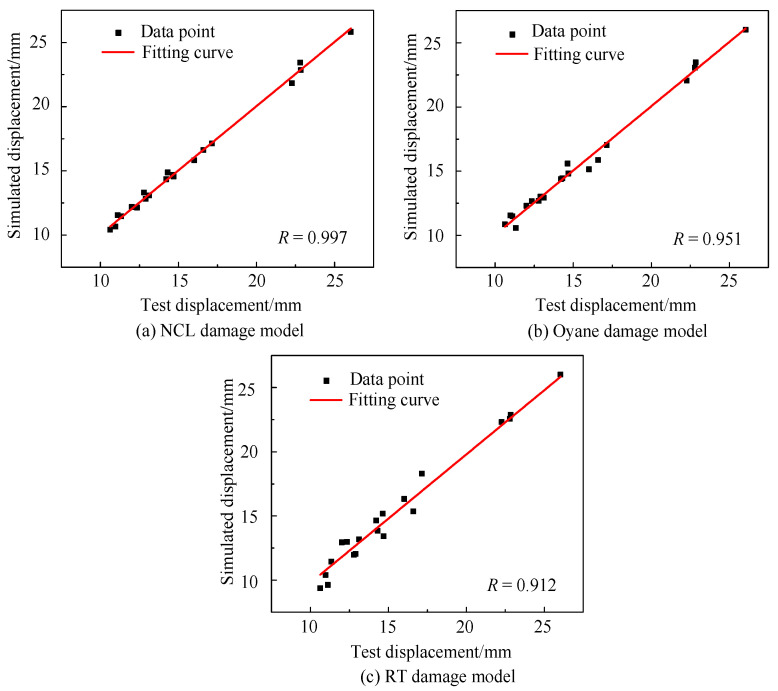
The correlation between simulation results and actual results.

Figure 11 shows the fracture displacement correlation between the simulated high-temperature stretching experiments based on NCL, Oyane, and RT high-temperature damage models, and actual hot stretching experiments. The predicted correlation coefficients of the NCL, Oyane, and RT models were 0.997, 0.951, and 0.912, respectively. It can be seen that all three models had high accuracy in predicting the Ti6Al4V alloy’s fracture failure. However, the NCL high-temperature damage model demonstrated higher prediction precision than both the Oyane and RT high-temperature damage models, by 4.6% and 8.5%, respectively. This is because material fracture occurs in locations under tensile stress, and the NCL damage model is suitable for the dominant form of fracture, which occurs under tensile stress [38], so it has a higher prediction accuracy. Therefore, the NCL high-temperature damage model is more suitable for predicting the thermal damage evolution of Ti6Al4V alloy.

## 6. Analysis of Hot Formability of Ti6Al4V Alloy Based on High-Temperature Damage Graph

**Figure 12:**
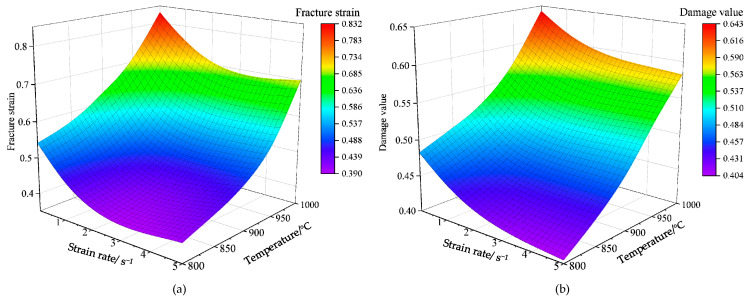
Damage surface of Ti6Al4V alloy: (**a**) Fracture strain graph; (**b**) Critical damage graph.

Figure 12a reflects the change in the Ti6AL4V alloy’s fracture strain in different conditions. The Ti6AL4V alloy’s fracture strain can be obtained using Equation (20) [39].

(20) $$\varepsilon_{f}=2\ln\left(\frac{a_{0}}{a_{f}}\right)$$

where $a_{0}$ is the specimen’s original diameter, and $a_{f}$ is the specimen’s diameter of the fracture surface after fracture.

Chen [40] concluded that the temperature and the strain rate have a major impact on the Ti6Al4V alloy’s fracture strain. As shown in Figure 12a, at 800 °C/5 s^−1^, the fracture strain’s minimum value was 0.422. When the temperature was constant and the strain rate reduces to 0.01 s^−1^, the fracture strain reaches 0.587. At 1000 °C/5 s^−1^, the fracture strain reached 0.733. At the temperature of 900 °C and with the strain rate changing from 5 s^−1^ to 0.01 s^−1^, the fracture strain increased from 0.482 to 0.614, with a change range of 0.132. Additionally, at 1000 °C, the fracture strain varied increasing from 0.733 to 0.739, with a change range of only 0.006. Therefore, at high temperatures, the impact of the temperature on fracture strain was greater than that of the strain rate. The lower the temperature, the greater the impact of the strain rate on fracture strain. This is because the increase in the β phase with better plasticity at higher temperatures results in higher plasticity of Ti6Al4V alloy [41].

Figure 12b shows the variation in the Ti6Al4V alloy’s critical damage value under different conditions. The larger the critical damage value, the lower the possibility that the Ti6Al4V alloy produces thermal cracks, and the better its thermal plasticity. The predictive trend of the damage model is consistent with the variation in the fracture strain. At 800 °C/5 s^−1^, the minimum critical damage value was 0.409. When the temperature was kept constant, and the strain rate was reduced to 0.01 s^−1^, the critical damage value increased to 0.501. Thus, at 800 °C, the strain rate decreased by 99.8%, and the hot-working plasticity of the material increased by 22.4%. At 1000 °C/0.01 s^−1^, the maximum critical damage value predicted using the NCL model was 0.664. Compared with the results obtained with a temperature of 800 °C and a strain rate of 0.01 s^−1^, the temperature increased by 25%, and the predicted thermal formability of the material using the damage model increased by 32.5%.

The results show that the fracture failure trend of TiAl4V alloy is positively correlated with temperature and negatively correlated with strain rate [42]. This indicates that increasing the temperature and decreasing the strain rate can improve the thermal formability of TiAl4V alloys and reduce the occurrence of damage cracks.

Under the different strain rates, the Ti6Al4V alloy’s critical damage value ranged from 0.580 to 0.664 at 1000 °C, and the change amplitude of the hot-working plasticity was approximately 14.4%. The Ti6Al4V alloy’s critical damage value ranged from 0.409 to 0.501 at 800 °C, and the change amplitude of the hot-working plasticity of the material was approximately 22.4%. Therefore, the strain rate’s impact on the critical damage value was relatively weak at higher temperatures. The strain rate’s effect on the critical damage value improved with the decrease in temperature. This result is the same as the trend observed for the fracture strain in Figure 12a. Thus, at higher temperatures, in the range of 800 °C to 1000 °C, the influence of the strain rate on hot formability becomes weaker for Ti6Al4V alloy.

## 7. Conclusions

In this paper, thermal tensile tests were carried out on Gleeble-1500D for Ti6Al4V alloy. The NCL, Oyane, and RT high-temperature damage models were established, considering the effect of the temperature and strain rate on damage evolution. The optimal parameters for the three damage models were obtained by combining the genetic algorithm and finite element methods. Finally, through program development, the high-temperature damage models were embedded into the Forge^®^ software, and the thermal tensile simulation tests were conducted. The correlation coefficient (R) between the stimulated fracture displacement and the actual fracture displacement was calculated to evaluate the predictive accuracy of the high-temperature damage models.

The results show that the force average cumulative error of the simulation and the actual test based on the NCL damage model was only 0.0175, demonstrating that the method of parameter inversion combined with the genetic algorithm has high accuracy in parameter identification. The predicted correlation coefficients (R) for the NCL, Oyane, and RT high-temperature damage models were 0.997, 0.951, and 0.912, respectively, with the NCL high-temperature damage model having the highest R-value. Therefore, the NCL high-temperature damage model established in this paper is more suitable for the hot-forming prediction of Ti6Al4V alloy.

Based on the NCL high-temperature damage model, the high-temperature damage graph of Ti6Al4V alloy was developed. Combined with the fracture strain graph, the correlation between the alloy’s thermal formability and the temperature and strain rate was determined.

The results indicate that the thermal formability of Ti6Al4V alloy exhibited a direct correlation with the temperature but an inverse correlation with the strain rate. At temperatures ranging from 800 to 1000 °C, the higher the temperature, the weaker the effect of the strain rate on the thermal formability of Ti6Al4V alloy.

## Figures and Tables

**Table 1:** The chemical compositions of Ti6Al4V (wt%).

Ti	Al	V	Fe	C	N	H	O
Bal.	6.11	3.93	0.131	0.016	<0.005	<0.001	0.113

**Table 2:** Constitutive model parameters of Ti6Al4V.

*A*	$m_{1}$	$m_{2}$	$m_{3}$	$m_{4}$	$m_{5}$	$m_{7}$	$m_{8}$	$m_{9}$
356.307	−0.00935	−0.032	−0.2007	−0.0033	0.00317	−2.356	0.00041	1.089

**Table 3:** NCL, Oyane, and RT damage models.

NCL	Oyane	RT
$C=\int_{0}^{{\bar{\varepsilon }}_{f}}\sigma_{1}/\bar{\sigma }d\bar{\varepsilon }$	$C=\int_{0}^{{\bar{\varepsilon }}_{f}}1+\alpha \sigma_{1}/\bar{\sigma }d\bar{\varepsilon }$	$C=\int_{0}^{{\bar{\varepsilon }}_{f}}\exp(\alpha \sigma_{m}/\bar{\sigma })d\bar{\varepsilon }$

In these equations,$\sigma_{1}$ is the first principal stress (MPa), $\bar{\sigma }$ is the equivalent stress (MPa), $\sigma_{m}$ is the average stress (MPa), $\bar{\varepsilon }$ is the equivalent strain, ${\bar{\varepsilon }}_{f}$ is the fracture strain, and α is a constant value.

**Table 4:** A modified form of NCL, Oyane, and RT damage models.

NCL	Oyane	RT
$K=\int_{0}^{{\bar{\varepsilon }}_{f}}\frac{\sigma_{1}/\bar{\sigma }}{S(Z)}d\bar{\varepsilon }$	$K=\int_{0}^{{\bar{\varepsilon }}_{f}}\frac{1+\alpha \sigma_{1}/\bar{\sigma }}{S(Z)}d\bar{\varepsilon }$	$K=\int_{0}^{{\bar{\varepsilon }}_{f}}\frac{\exp(\alpha \sigma_{m}/\bar{\sigma })}{S(Z)}d\bar{\varepsilon }$

**Table 5:** The parameters of genetic algorithm.

Population Size	Crossover Rate	Mutation Rate	Target Function Value	Maximum Iterations
30	0.7	0.04	0.02	120

**Table 6:** Critical damage values of each damage criterion under different conditions.

Damage Criterion	Strain Rate	800 °C	850 °C	900 °C	950 °C	1000 °C
NCL	0.01 s^−1^	0.501	0.512	0.551	0.574	0.664
	0.1 s^−1^	0.471	0.503	0.508	0.556	0.636
	1 s^−1^	0.449	0.453	0.480	0.545	0.613
	5 s^−1^	0.409	0.437	0.466	0.549	0.580
Oyane	0.01 s^−1^	0.469	0.478	0.494	0.513	0.692
	0.1 s^−1^	0.401	0.417	0.414	0.435	0.681
	1 s^1^	0.367	0.387	0.358	0.426	0.678
	5 s^−1^	0.302	0.335	0.384	0.421	0.642
RT	0.01 s^−1^	0.524	0.523	0.612	0.581	0.645
	0.1 s^1^	0.491	0.484	0.504	0.573	0.644
	1 s^−1^	0.435	0.436	0.456	0.524	0.623
	5 s^−1^	0.415	0.437	0.476	0.534	0.610

**Table 7:** The average cumulative error of damage mode.

Damage Criterion	NCL	Oyane	RT
**Average cumulative error**	0.0175	0.0192	0.0168

**Table 8:** Zener–Hollomon coefficients of Ti6Al4V alloy at different conditions.

T/°C	$\dot{\varepsilon }$/s^−1^	ln^Z^
800	0.01	86.09553
850	0.01	82.05721
900	0.01	78.36316
950	0.01	74.97116
1000	0.01	71.84562
800	0.1	88.39811
850	0.1	84.35979
900	0.1	80.66574
950	0.1	77.27374
1000	0.1	74.1482
800	1	90.7007
850	1	86.66238
900	1	82.96833
950	1	79.57633
1000	1	76.45079
800	5	92.31014
850	5	88.27182
900	5	84.57777
950	5	81.18577
1000	5	78.06022

## Data Availability

The data presented in this study are available on request from the corresponding author. The data are not publicly available due to these data are part of ongoing research.
